# Widespread changes in mRNA stability contribute to quiescence-specific gene expression patterns in a fibroblast model of quiescence

**DOI:** 10.1186/s12864-017-3521-0

**Published:** 2017-02-01

**Authors:** Elizabeth L. Johnson, David G. Robinson, Hilary A. Coller

**Affiliations:** 1 Department of Molecular Biology, Princeton University, Princeton, NJ 08544 USA; 2 Lewis-Sigler Institute for Integrative Genomics, Princeton University, Princeton, NJ 08544 USA; 3 Department of Molecular, Cell, and Developmental Biology, University of California, Los Angeles, CA 90095 USA; 4 Department of Biological Chemistry, David Geffen School of Medicine, Los Angeles, CA 90095 USA

**Keywords:** Quiescence, mRNA stability, *miR-29*, Gene expression, Post-transcriptional regulation, Cell-cycle

## Abstract

**Background:**

Quiescence, reversible exit from the cell division cycle, is characterized by large-scale changes in steady-state gene expression, yet mechanisms controlling these changes are in need of further elucidation. In order to characterize the effects of post-transcriptional control on the quiescent transcriptome in human fibroblasts, we determined mRNA decay rates for over 10,000 genes using a transcription shut-off time-course.

**Results:**

We found that ~500 of the genes monitored exhibited significant changes in decay rate upon quiescence induction. Genes involved in RNA processing and ribosome biogenesis were destabilized with quiescence, while genes involved in the developmental process were stabilized with quiescence. Moreover, extracellular matrix genes demonstrated an upregulation of gene expression that corresponded with a stabilization of these transcripts. Additionally, targets of a quiescence-associated microRNA (*miR-29*) were significantly enriched in the fraction of transcripts that were stabilized during quiescence.

**Conclusion:**

Coordinated stability changes in clusters of genes with important functions in fibroblast quiescence maintenance are highly correlated with quiescence gene expression patterns. Analysis of *miR-29* target decay rates suggests that microRNA-induced changes in RNA stability are important contributors to the quiescence gene expression program in fibroblasts. The identification of multiple stability-related gene clusters suggests that other posttranscriptional regulators of transcript stability may contribute to the coordination of quiescence gene expression. Such regulators may ultimately prove to be valuable targets for therapeutics that target proliferative cells, for instance, in cancer or fibrosis.

**Electronic supplementary material:**

The online version of this article (doi:10.1186/s12864-017-3521-0) contains supplementary material, which is available to authorized users.

## Background

Cellular quiescence is a state of cell cycle arrest that is characterized by the unique ability of cells to exit and re-enter the cell division cycle upon presentation of the appropriate stimulus. The proliferation of B-cells during an immune response, hepatic stellate cells in response to liver injury, and skin fibroblasts during wound healing all rely on the ability of cells to properly re-enter the cell cycle from a quiescent state [1–3]. Upon induction to the quiescent state, there are well-documented changes in gene expression, but the regulation and coordination of gene expression changes with quiescence is not fully understood [4–6]. Transcriptional repressors such as HES1 and FOXC1 that can control the expression of clusters of genes involved in maintaining the reversibility of quiescence have been shown to account for some of these observed expression changes [5, 7, 8]. In addition to transcriptional control, post-transcriptional contributions to quiescence gene regulation have the potential to have large effects on the quiescence gene expression signature. During quiescence, downregulation of cell cycle progression-promoting genes such as the transcription factor MYC is important for sustaining cell cycle exit. A transcript-stabilizing truncation containing an AU-rich element (ARE) in the MYC 3’UTR can lead to cell cycle misregulation and oncogenic transformation [9]. Moreover, RNA binding proteins that elicit decay through interactions with AREs have been shown to be necessary in maintaining lymphocyte quiescence [10]. Additionally, upregulation of cell cycle progression inhibitors like the cyclin-dependent kinase inhibitor CDKNIB (p27 (Kip1)) is required for cell cycle exit and CDKN1B has been shown to be extensively post-transcriptionally regulated [11, 12].

Post-transcriptional regulatory mechanisms are emerging as major contributors to the biological functions essential to quiescence biology. Directly determining the stability of mRNA transcripts has multiple advantages over steady-state determinations of gene expression for understanding contributions of post-transcriptional control to gene regulation. One advantage is that RNA stability measurements increase the ability to identify clusters of genes that are controlled by a common post-transcriptional regulator [13, 14]. In a lymphocyte model of quiescence, it was shown that around 50% of the gene expression changes observed upon activation to proliferation were controlled at the level of RNA stability [15, 16]. Additionally, microRNAs, which can act as posttranscriptional negative regulators of gene expression, have been shown to have effects on transcript stability [17]. Thus, clustering transcripts by changes in stability can give greater resolution to the identification of miRNAs important for stability-based changes in gene expression rather than using steady state gene expression measurements for these inferences. Coupling stability measurements with steady-state gene expression measurements gives more information on how the steady state was reached and how future perturbations may affect the potential to reach a new steady state. This information is lost when solely analyzing gene expression data [18].

In this study, we characterized changes in mRNA stability between proliferating and quiescent fibroblasts using genome-wide measurements of mRNA stability. Analysis of these measurements revealed stability-regulated gene clusters with shared biological functions. Concurrent measurements of steady-state gene expression allowed more insight into how up and down regulated gene clusters were affected by changes in RNA stability. Notably, a stability-regulated cluster was defined by targets of the quiescence-associated miRNA, *miR-29,* giving further insight into its mechanism of action.

## Results

### Genome-wide changes in RNA stability with quiescence induction

To understand the global role of post-transcriptional control in the regulation of the quiescent transcriptome, we used microarrays to determine half-lives of over 10,000 transcripts in human foreskin fibroblasts. Transcription was inhibited in either proliferating (P) cells or cells made quiescent by 7 days of contact inhibition of growth (CI7). Samples were collected over an 8 h time period and analyzed by microarray. Decay constants were calculated by fitting the log decrease in transcript abundance to a linear model and genes with a poor fit to the linear model were filtered from further analysis (see Methods). Correlations of decay constants with publically available data sets were in good concordance with previous calculations in other cell culture models (Additional file 1).

**Fig. 1:**
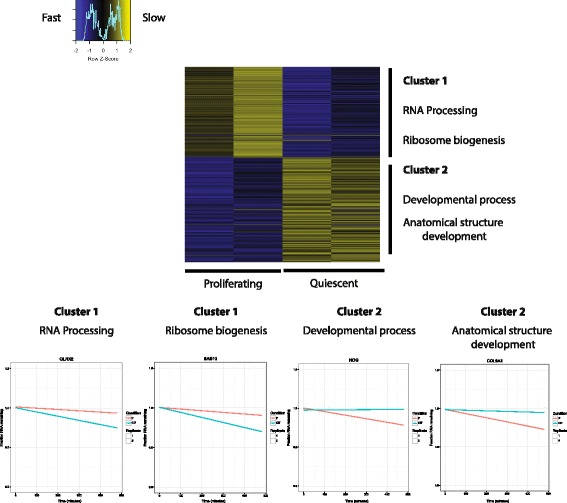
Genes that change in RNA stability with quiescence are related in function. Heatmap showing the changes in RNA stability between proliferating (P) and 7-day contact inhibited fibroblasts (CI7). Columns 1 and 2 are biological replicates of mean centered decay rate constant determinations in P fibroblasts and columns 3 and 4 are replicates of decay rate constant determinations in CI7 fibroblasts. Positive values (*yellow*) indicate slower decay compared to the mean rate for that gene while negative values (*blue*) indicate that genes have faster decay compared to the mean rate for that gene. Values were clustered into two unique groups using the k-means algorithm. Gene ontology terms that are significantly enriched in a cluster are marked to the right of the heatmap. Decay profiles of representative genes from each cluster are displayed below the heatmap

To identify differentially post-transcriptionally regulated genes between P and CI7 fibroblasts, we focused on the 485 genes that had significant changes in transcript stability between the two states (Fig. 1a). Genes were identified as having significantly different decay rates if time course fluorescence intensities had a significantly different slope, according to an ANOVA F-test, comparing P and CI7 conditions (FDR < 0.05, see Methods).

K-means clustering of decay constants across time course replicates resulted in two main profiles of stability changes (Fig. 1a). Genes in cluster 1 exhibited faster decay during quiescence and were enriched for genes involved in RNA processing and ribosome biogenesis. Genes in cluster 2 exhibited faster decay during proliferation and were enriched in genes involved in developmental processes and anatomical structure development.

Genes with no significant change in RNA stability measurements between P and CI7 fibroblasts were also characterized by enrichments in Gene Ontology (GO) terms. The GO terms identified shared similarity to those reported in previous studies of transcript decay rates in human cells [19, 20]. Fast-decaying transcripts in both states were enriched for GO terms such as “regulation of gene expression”, while slow-decaying transcripts in both states were enriched for ontology terms related to “cellular respiration”. A full list of clustered decay rate distributions and corresponding enriched GO terms is included in Additional file 2, Additional file 3: Figure S1, and Additional file 4. Gene set enrichment analysis of genes with significant changes in RNA stability provided further resolution of stability-regulated quiescence gene sets. Top sets with faster decay during quiescence included mitochondrial translation, DNA damage response, and RNA-related processes (Additional file 3: Figure S2A). In contrast, top sets with slower decay during quiescence included genes involved in differentiation and extracellular matrix disassembly (Additional file 3: Figure S2B).

### Changes in RNA stability correlate with a subset of gene categories that have large changes in gene expression during quiescence

**Fig. 2:**
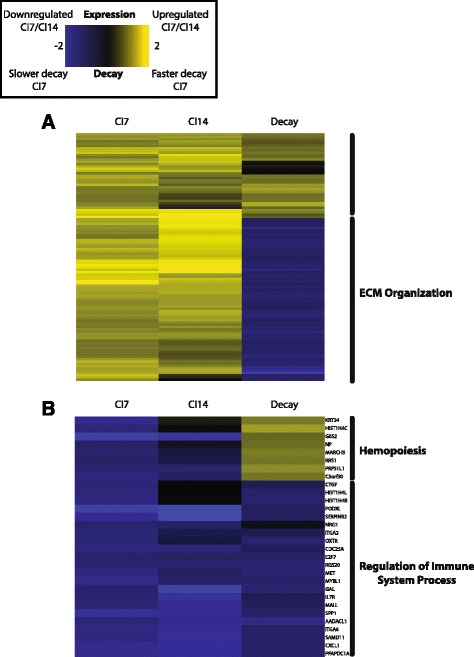
Gene expression and RNA stability heatmap for the top upregulated **a** and downregulated **b** genes with quiescence. Columns 1 and 2 are the log_2_ fold change in gene expression with quiescence $\left({ \log}_2\left(\frac{CI7 Abundance}{PAbundance}\right)\right)$ from microarray gene expression profiling of 7-day contact inhibited (CI7) and 14-day contact inhibited (CI14) fibroblasts. Column 3 is the log difference in decay constants (*K*_*D*_ *proliferation* − *K*_*D*_ *quiescence*) shrunken by the local false discovery rate (see Methods) scaled to fit within the bounds of the gene expression values between CI7 and P fibroblasts. Gene ontology terms that are significantly enriched in a cluster are marked to the right of the heatmap

In order to better understand how these genome-wide changes in RNA stability are associated with gene expression, we looked for correlations between mRNA stability changes and changes in steady-state gene expression upon quiescence induction. We profiled gene expression in P versus CI7 fibroblasts using Agilent two-color microarrays to understand the contribution of RNA stability to changes in gene expression. There was no overall correlation between changes in expression and stability of the genes common between the two datasets. However, there were groups of genes that had well-defined changes in stability that were correlated with large changes in steady-state gene expression levels. Notably, the majority of the most strongly upregulated genes in the quiescent state $\left({ \log}_2\left(\frac{CI7 Abundance}{PAbundance}\right)>2\right)$ were preferentially stabilized with quiescence (Fig. 2a). These genes were enriched for extracellular matrix (ECM) organization genes. Included in this cluster of genes are multiple genes that encode collagens including COL14A1 and COL15A1, which have well-defined roles in ECM creation and can be overexpressed during fibrosis [21–23]. Among the most strongly downregulated genes in the quiescent state $\left({ \log}_2\left(\frac{CI7 Abundance}{Pabundance}\right)<-2\right)$, the majority of genes were also preferentially stabilized during quiescence (Fig. 2b). These genes expressed at lower levels in proliferating cells and exhibiting more rapid decay in proliferating cells included the critical cell cycle regulator CDC25A.

### *miR-29* targets involving extracellular matrix are stabilized during quiescence

**Fig. 3:**
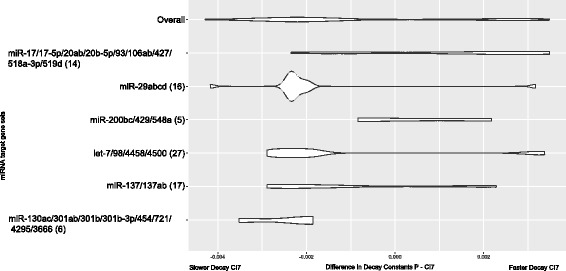
miRNAs with targets enriched for differential stability between proliferating and 7 day contact-inhibited fibroblasts. Violin plots display the distribution of the difference in decay rates for the targets of miRNAs with significant enrichment of stabilized or destabilized targets with quiescence. The difference in decay constants between P and CI7 fibroblasts (P_constant_ – CI7_constant_) is displayed on the x-axis. The number of transcripts in the distribution is displayed in parentheses after the miRNA family name on the y-axis

**Fig. 4:**
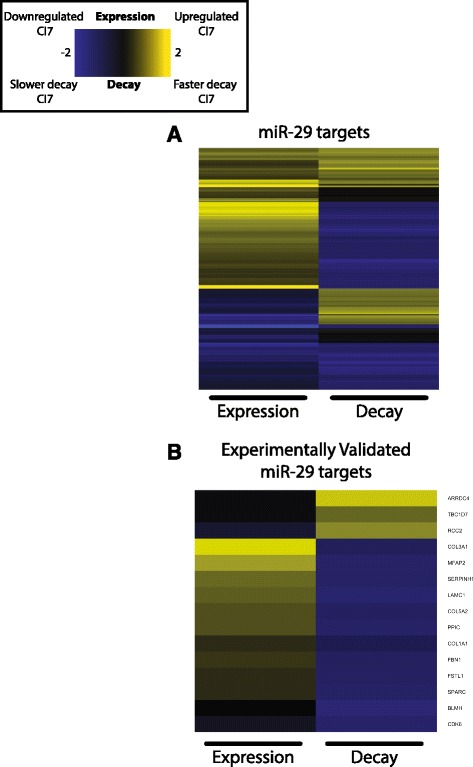
Gene expression and stability change heatmap for collagen-related *miR-29* targets. Gene expression and RNA decay constant changes for computationally predicted **a** and experimentally validated **b** *miR-29* targets. *Black bars* on the bottom of the heatmap mark the columns that are changes in gene expression while the last column is the log of the change in decay constant between P and CI7 fibroblasts. Gene expression columns show the log_2_ fold change in gene expression between P and CI7 fibroblasts $\left({ \log}_2\left(\frac{CI7 Abundance}{PAbundance}\right)\right)$. Decay columns are the log difference in decay constants (*K*_*D*_ *proliferation* − *K*_*D*_ *quiescence*) shrunken by the false discovery rate scaled to fit within the bounds of the gene expression values between CI7 and P fibroblasts (see Methods) between CI7 and P fibroblasts. Gene names are to the right of the heatmap

To identify potential regulators responsible for a coordinated change in the stability and expression of genes, we used predicted miRNA targets from TargetScan [24] as gene set references and identified gene sets for which the differences in corrected decay constants (see Methods) between conditions were different for the genes in each miRNA target set as compared to genes outside of the target set [25]. This analysis allowed us to identify miRNA targets enriched in categories of genes that were stabilized or destabilized in the quiescent state (Fig. 3). Out of the 485 differentially stabilized transcripts, mRNA targets of *miR-29*, *let-7*, *miR-137*, and *miR-130* were stabilized with quiescence while *miR-17* and *miR-200* targets were stabilized with proliferation. We decided to focus on *miR-29* targets based on our previous demonstration that *miR-29* plays an important functional role in quiescence [26]. *miR-29* targets are significantly stabilized with quiescence, and are enriched for genes that encode proteins that are found in the ECM or are involved in ECM remodelling. In our previous study, targets of the *miR-29* family were more likely to change in abundance with quiescence than the targets of any other microRNA investigated [26]. Downregulation of *miR-29* during quiescence resulted in a relief of negative regulation of these *miR-29* targets, and thus, they are expressed at higher levels in quiescent than proliferating cells. In this current analysis, targets of the *miR-29* family (miR-29abcd) were significantly more stable during quiescence (*χ* ^2^, 1, *p* < 0.05). The decay and gene expression profiles of computationally-predicted *miR-29* collagen-related targets show a strong pattern of stabilization of *miR-29* targets in the quiescent state, and higher expression of the associated genes in the quiescent compared with the proliferating state (Fig. 4a). Since miRNA target prediction can include many false positives and targets that are only regulated in specific biological contexts, we refined this target set even further by focusing on the specific transcripts that we found to be regulated when *miR-29* was introduced into primary human dermal fibroblasts [26]. We observed that experimentally-validated *miR-29* targets exhibited an even stronger gene expression-stability signature of upregulation of gene expression and transcript stabilization with quiescence than the set of computationally predicted targets (Fig. 4b). In summary, *miR-29* levels decrease with quiescence and this relief of negative regulation correlates with our observation of increased stability of *miR-29* targets. By monitoring transcript decay rates, we can now conclude that the observed *miR-29* regulation of quiescence targets reflects changes in mRNA stability.

## Discussion

In a fibroblast model of quiescence, we observed that RNA stability, an important mechanism for post-transcriptional regulation, changes on a genome-wide scale during quiescence. Defining whether the expression levels of a transcript are controlled by transcriptional or post-transcriptional mechanisms can provide further insight into the mechanisms underlying coordinated changes in gene expression. Our findings are consistent with other reports that transcript stability can be an important contributor to changes in cell fate [14, 27, 28]. For instance, in differentiating C2C12 cells, there were changes in transcript decay during differentiation to a myoblast phenotype that were closely associated with changes in gene expression [27]. Additionally, in lymphocytes, transcript decay rates were shown to be critical for the sequential waves of gene expression events that occur upon activation [28]. In our study of proliferating versus quiescent fibroblasts, there was no overall correlation between mRNA abundance and decay rate. With a more focused analysis on genes with the most significant increase or decrease in expression, we were able to determine that extracellular matrix genes were characterized by slower decay rates and a higher abundance in quiescent compared with proliferating cells.

Extracellular matrix metabolism genes, which are highly upregulated during quiescence, exhibit increased transcript stability in the quiescent state compared to the stability of these same genes when fibroblasts are proliferating. Such findings would be consistent with an important role for skin fibroblasts in synthesizing collagen that forms the connective tissue in skin. These data suggest that the changes in collagen and extracellular matrix production are controlled, at least in part, post-transcriptionally at the level of mRNA stability. This insight into gene regulation is masked when profiling steady state levels of gene expression, but when RNA stability is also profiled, more insight into the mechanistic coordination of related gene sets is gained. Moreover, *miR-29*, a negative regulator of collagen gene expression, is downregulated during quiescence induction. Upregulation of *miR-29* during proliferation leads to negative regulation of these same transcripts, which share a common biological function, by decreasing their stability.

Global changes in miRNA abundance have been documented in multiple mammalian quiescence models [26, 29, 30] and contribute to regulatory networks necessary for defining and maintaining cell cycle phenotypes [31, 32]. Differences in corresponding RNA binding protein complexes [33] and transcript architecture [34] during quiescence can lead to different mechanisms of miRNA-dependent transcript regulation. Cell-cell contact was reported to enhance miRNA processing, resulting in increased miRNA biogenesis and more efficient formation of RNA-induced silencing complexes [29]. When considering miRNA-dependent effects on RNA stability, this model suggests that there might be rapid decay of miRNA targets in quiescent cells. In another study, miRNAs have been reported to be stored in inactive low molecular weight Argonaute protein complexes that lack GW182 in a quiescence model [33]. These findings suggest that miRNA target genes might be more stable in the quiescent state. Our data identified examples of both cases where a portion of miRNA targets were more stable during quiescence (including *miR-29* targets), and a portion of targets were less stable during quiescence. These results are consistent with our own analysis in which the levels of miRNAs were monitored by microarray and *miR-29* levels were discovered to decline relative to other miRNAs in quiescent cells [26]. The results are also consistent with our findings that *miR-29* hastens cell cycle re-entry from quiescence [26].

miRNA-dependent alterations of transcript stability [17, 35, 36] and translational efficiency [37] have both been identified as contributors to gene regulatory changes and this work adds to our understanding of the role of miRNAs in the regulation of transcript stability changes between cell cycle states. Our analysis of miRNA targets enriched for differential decay between P and CI7 fibroblasts highlight a potential role for the *miR-17-92* cluster, and *miR-200* in promoting transcript decay in quiescent cells, and *miR-130* in promoting transcript decay in proliferating cells. Moreover, the *miR-17-92 cluster* in particular has been previously implicated in cell cycle regulation [38, 39]. These microRNAs are candidates for further study as potential regulators of the proliferation-quiescence transition.

Our work further defines a role for specific changes in transcript stability as contributors to gene regulation in a fibroblast quiescence model. Regulators of gene expression such as *miR-29* serve as candidate targets for affecting the expression of extracellular matrix expression, for instance, in fibrotic disease.

## Conclusion

This study provides a rich set of data to add to the growing knowledge about how mRNA stability contributes to gene expression changes as a cell responds to various stimuli. We were able to integrate data on mRNA transcript stability, mRNA transcript abundance and miRNA expression to better understand how genes involved in extracellular matrix metabolism are regulated during fibroblast quiescence. Further studies elucidating the role of RNA biology in fibroblast quiescence will improve our understanding of complex proliferative and secretory diseases such as fibrosis.

## Methods

### Cell culture and transcription shutoff time course

Human fibroblasts were isolated from the dermal layer of neonatal foreskin tissue as previously described [40]. Proliferating and contact-inhibited fibroblast were maintained in DMEM supplemented with 10% fetal bovine serum (FBS). Proliferating cells were seeded at 5 × 10^5^ cells per 10 cm plate and split every 48 h. Contact-inhibited cells were seeded at 5 × 10^5^ cells per 10 cm plate with medium changes every 48 h until the end of a 7-day time period.

To inhibit transcription in proliferating and 7-day contact-inhibited fibroblasts, actinomycin D was added to the culture media at a concentration of 15 μg/mL. Cells were washed with PBS and cell lysates were collected using Trizol (Life Technologies) at 0, 120, 240, and 480 min after addition of actinomycin D.

### Decay rate constant calculations

Based on the first order nature of mRNA decay kinetics, fluorescence intensities were log-transformed and fit to a linear decay model, using time, cell cycle condition (P or CI7), and biological replicate as predictors. Genes with a poor fit to the log linear decay model (6297 of 14212 total transcripts, ANOVA F-test, *p* > 0.05) were filtered from subsequent analysis. Transcripts with a significant interaction term between condition and time, according to an ANOVA F-test (FDR < 0.05), were considered to have different decay rates between P and CI7 fibroblasts. To aide in the interpretation of changes in decay between states, a metric (decay metric) to compare P to CI7 decay constants was calculated by taking the difference between the P and CI7 decay constants and using the local False Discovery Rate [41] to bring the value of constant comparisons without a significant change between conditions towards 0. All original intensities and calculated values are available in Additional file 4.

### Microarray labeling, hybridization, and raw data processing

RNA was isolated from Trizol lysates according to the manufacturer’s protocol [42]. RNA quality was verified on a Bioanalyzer 2100 using reagents from the RNA nano 6000 kit (Agilent Technology). RNA that passed quality and yield cutoffs were reverse transcribed into cDNA and subsequently labelled with the dye Cyanine-3 through the transcription of cRNA using the Quick Amp Microarray Labelling Kit (Agilent Technology). RNA spike-in control A (Agilent Technology) was added to each RNA sample in the time course at equal amounts as a control for labelling and hybridization efficiency. Labeled cRNA was hybridized to Human 4 × 44 K gene expression microarrays using the one-color format for 17 h at 65 °C (Agilent Technology). Fluorescence intensities were detected using a microarray scanner (Agilent Technology) and assigned to the appropriate gene using Feature Extractor 6.1 (Agilent Technology) software. Probes with fluorescent intensities above background and above Feature Extractor quality control thresholds were used in decay rate constant determinations.

### Microarray cRNA transcription and hybridization

Total RNA samples from P, CI7, and CI14 cells were reverse transcribed into cDNA and fluorescently labeled with Cyanine 3-CTP (CY3) (quiescent samples) or Cyanine 5-CTP (CY5) (proliferating samples) to make cRNA according to the manufacturer’s protocol for the Quick Amp Labeling Kit for Microarray Analysis (Agilent). cRNA samples that passed yield and labeling standards were fragmented and proliferating and quiescent samples were hybridized to Human gene expression 4 × 44 K arrays (Agilent) for 17 h at 65 °C in an oven rotating the arrays at 10 rpm.

Fluorescence intensities were detected using the Genepix scanner (Agilent) and probe identities were determined using Agilent’s feature extractor version 11.5. Probes detected over background fluorescence thresholds were used in subsequent gene expression analysis. Microarray data was uploaded to the Princeton University MicroArray database (PUMAdb) and is accessible for download with the proper permissions. Log_2_ ratios of quiescent versus proliferating gene expression are available in Additional file 5.

### Gene ontology analysis and miRNA enrichment analysis

Overrepresentation of gene ontology terms in gene clusters defined by k-means clustering of decay constants was determined using the Lewis-Sigler Gene Ontology tool (http://go.princeton.edu/). miRNA enrichment was calculated by collecting the predicted TargetScan [24] targets of each miRNA, then using a Wilcoxon rank-sum test to compare differences in decay constants P_constant_ – CI7_constant_ within the set to genes outside the set [25]. Enrichment tests and violin plots displaying the differences in decay constants P_constant_ – CI7_constant_ for targets of each miRNA were performed using the GSEAMA package (David Robinson – Princeton University) implemented in R. The difference in decay constants between P and CI7 fibroblasts from *miR-29* targets were compared to non-targets using a Chi-squared test for independence to test whether miR-29 targets versus non-targets had a higher proportion of genes that decayed faster during proliferation as compared to quiescence.

## Additional files

Additional file 1: Comparison of decay constant calculations between genome wide studies of RNA decay. (DOCX 99 kb)
Additional file 2: Gene ontology tables of genes with fast or slow decaying genes in both proliferating and quiescent fibroblasts. (XLSX 69 kb)
Additional file 3: Figure S1A. Example decay profiles of stable (ACTB) and unstable (JUN) transcripts in both the proliferating and 7-day contact inhibited state. **Figure S1B.** Decay constant distributions comparing proliferating (red) versus 7-day contact inhibited fibroblasts (green) separated by k-means clusters. **Figure S2A.** Gene set enrichment analysis of genes destabilized with quiescence. **Figure S2B.** Gene set enrichment analysis of genes stabilized with quiescence (2.7 mb). (PDF 2619 kb)
Additional file 4: Decay time course microarray fluorescence intensities, calculated decay constants, significance testing and k-means clustering results. (XLSX 7664 kb)
Additional file 5: Microarray determined gene expression values for proliferating versus 7-day contact inhibited or 14-day contact inhibited fibroblasts. (XLSX 1119 kb)
