# Supplementary material for: Widespread changes in mRNA stability contribute to quiescence-specific gene expression patterns in a fibroblast model of quiescence
Source: BMC Genomics. 2017 Feb 1;18:123. doi: 10.1186/s12864-017-3521-0 (PMC5286691; doi:10.1186/s12864-017-3521-0)
Supplement: Additional file 1: — Comparison of decay constant calculations between genome wide studies of RNA decay. (DOCX 99 kb) [file 12864_2017_3521_MOESM1_ESM.docx]

**Additional File 1**

**Decay rate validation**

| Dataset 1 | Dataset 2 | Pearson correlation for genes with half lives under 500 minutes (R_p_) | Coefficient of determination - R^2^ (R_s_) |
| --- | --- | --- | --- |
| This study | Neff et. al. (2012) [1] | 0.47 | 0.22 |
| This study | Friedel et. al. (2009) [2] | 0.39 | 0.15 |
| Neff et. al. (2012) [1] | Friedel et. al. (2009) [2] | 0.18 | 0.03 |

Correlation between biological replicates in our data set:

Proliferation biological replicates – r_p_=0.86, r_s_ = 0.74, n=15920

CI7 biological replicates – r_p_=0.75, r_s_= 0.56, n=15920

To understand how our decay constant determinations align with previous calculations and protocols in the literature, we compared the half-lives determined in this study to publically available data from two relevant studies performed in human cell lines. One study used actinomycin D to block transcription and measured transcript decay in human fibroblasts [1], while the other used metabolic labeling to determine transcript half-lives by calculating ratios of newly transcribed to pre-existing RNA in human B-cells [2]. For this analysis, we focused on transcripts with half-lives within the range of accuracy for shortest time course (under 500 minutes). Our data was most correlated with the Neff et. al. data set (r = 0.47) as compared to the Friedel et. al. data set (r = 0.39) and was more highly correlated with the other data sets than when comparing the Neff et al. and Friedel et al. datasets to each other (r = 0.18). Additionally, the correlation between two biological replicates of decay rate calculations in both proliferating (r_p_=0.86, r_s_ = 0.74, n=15920) and 7dCI (r_p_=0.75, r_s_= 0.56, n=15920) conditions show reproducibility at levels comparable to similar studies in the literature [3].

**References**

1. Neff AT, Lee JY, Wilusz J, Tian B, Wilusz CJ: **Global analysis reveals multiple pathways for unique regulation of mRNA decay in induced pluripotent stem cells**. *Genome Res* 2012, **22**(8):1457-1467.

2. Friedel CC, Dolken L, Ruzsics Z, Koszinowski UH, Zimmer R: **Conserved principles of mammalian transcriptional regulation revealed by RNA half-life**. *Nucleic acids research* 2009, **37**(17):e115.

3. Spies N, Burge CB, Bartel DP: **3' UTR-isoform choice has limited influence on the stability and translational efficiency of most mRNAs in mouse fibroblasts**. *Genome Res* 2013.
