# Supplementary figures and images for: Widespread changes in mRNA stability contribute to quiescence-specific gene expression patterns in a fibroblast model of quiescence
Source: BMC Genomics. 2017 Feb 1;18:123. doi: 10.1186/s12864-017-3521-0 (PMC5286691; doi:10.1186/s12864-017-3521-0)

Supplement Fig. 1

A

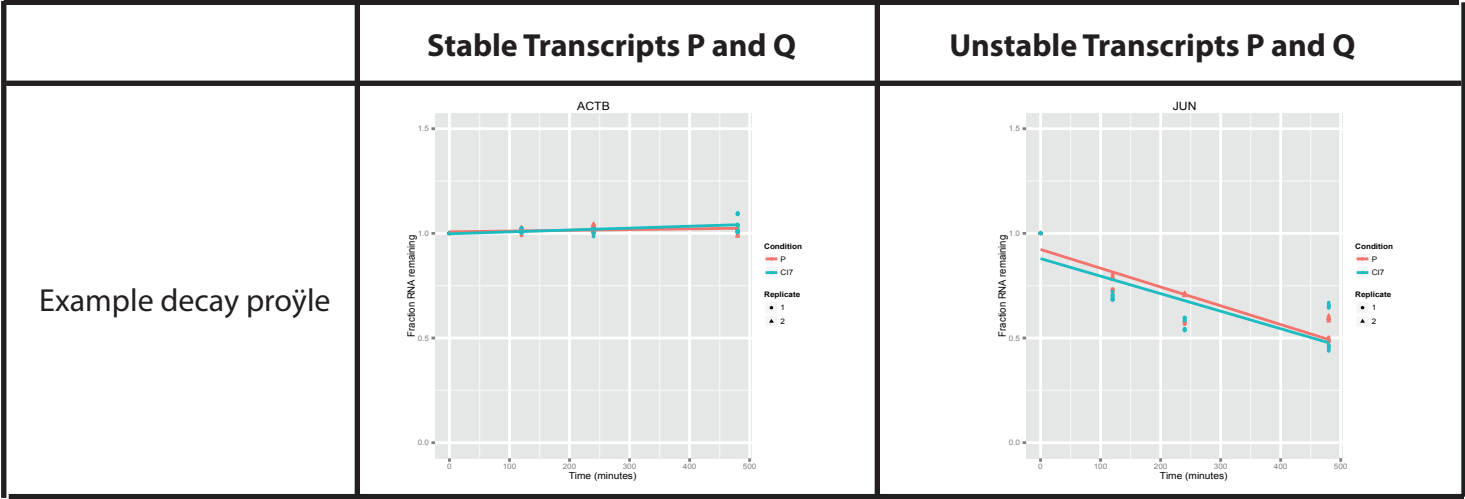

B

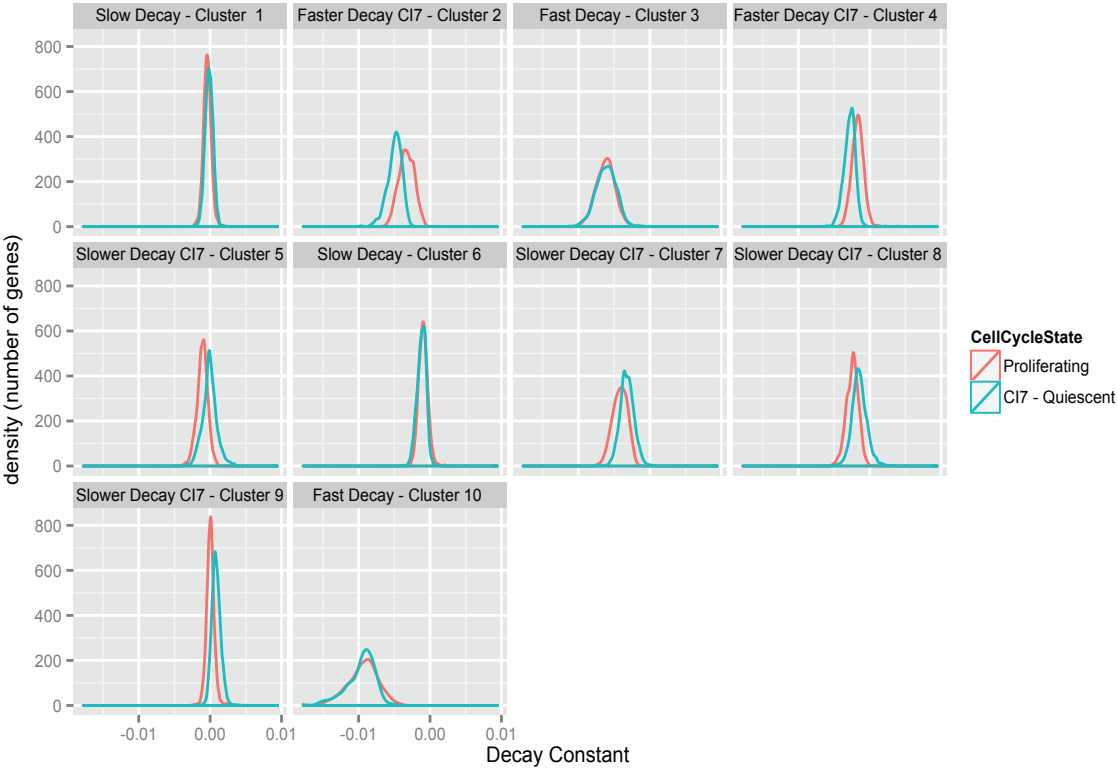

Supplement Fig. 2A

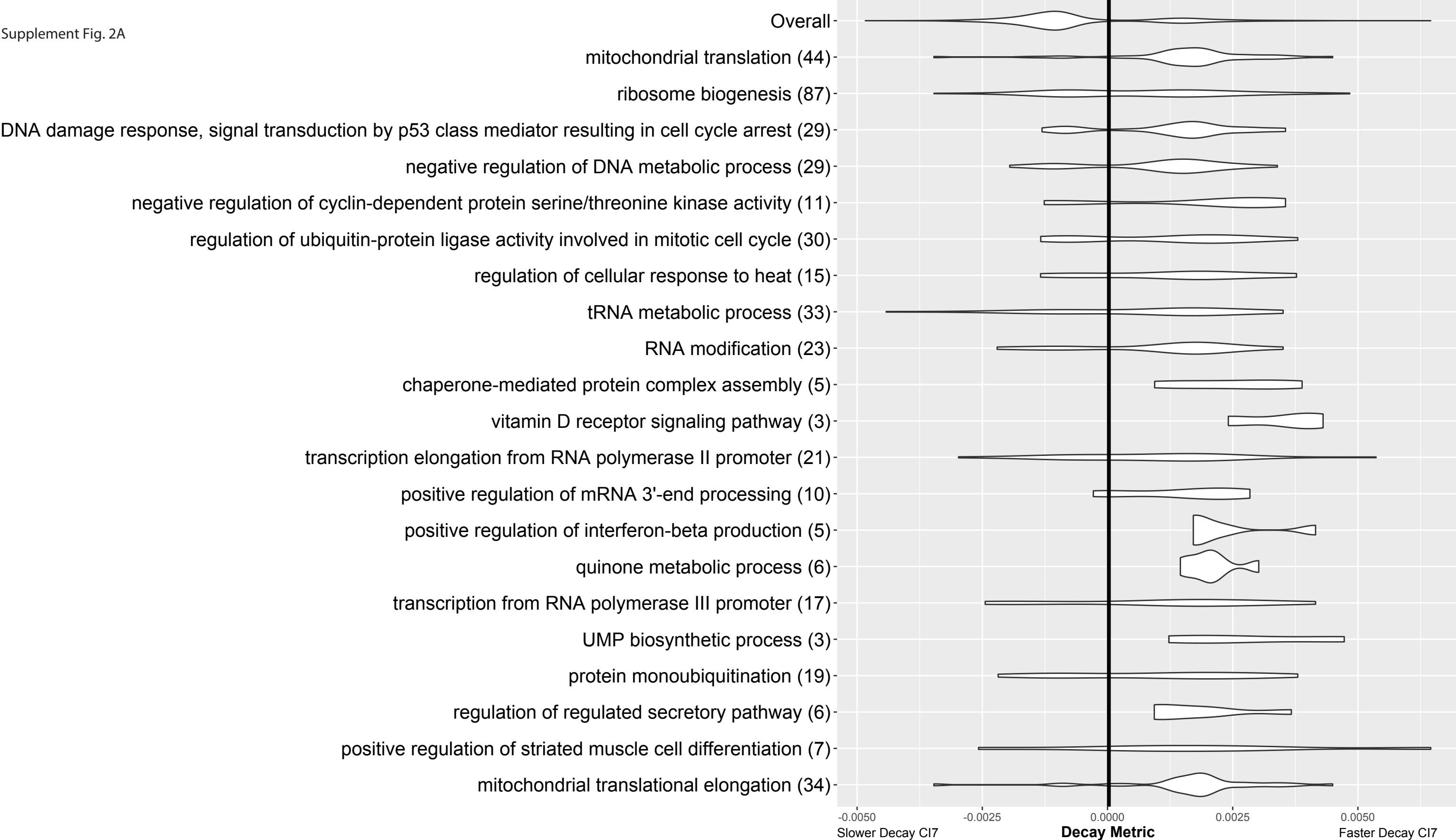

Supplement Fig. 2B

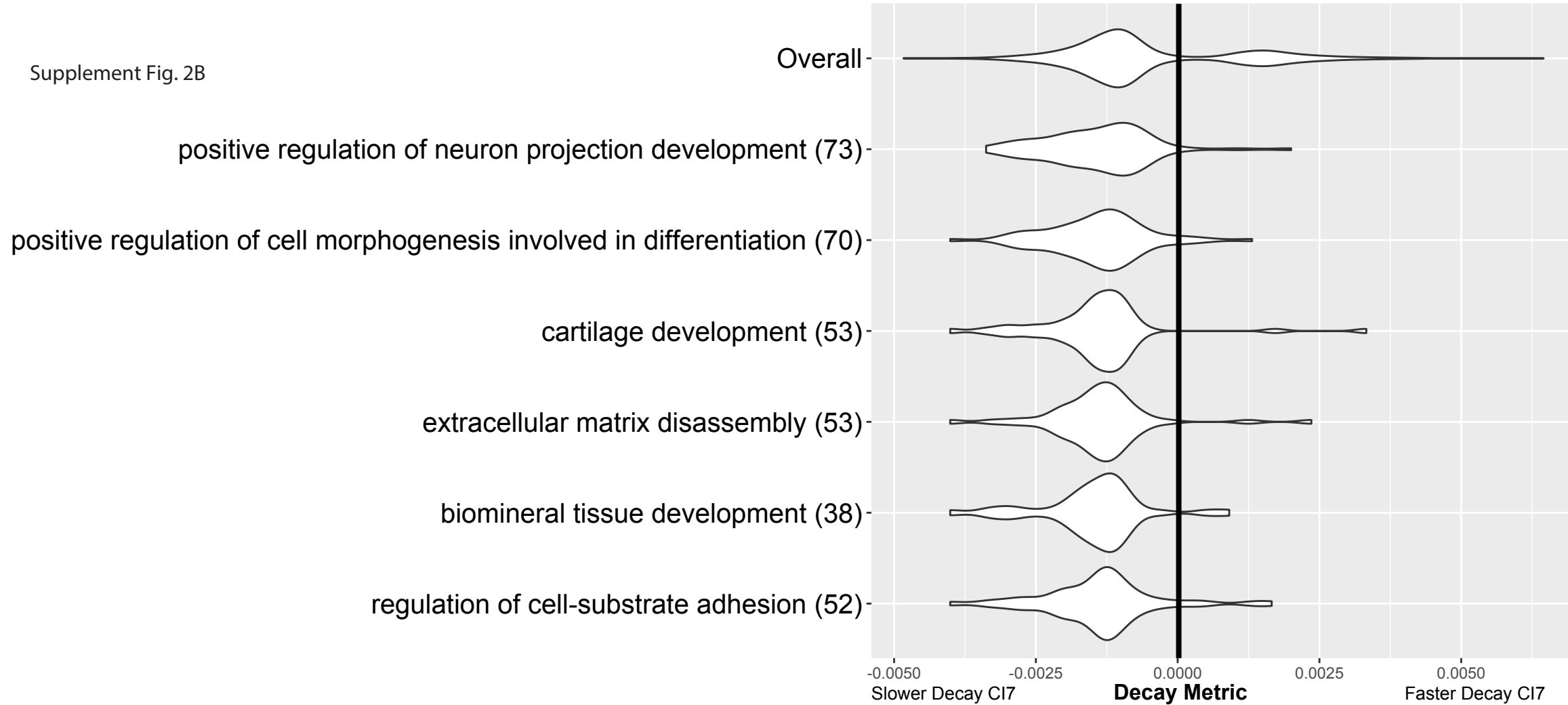

Supplement: Additional file 3: Figure S1A. — Example decay profiles of stable (ACTB) and unstable (JUN) transcripts in both the proliferating and 7-day contact inhibited state. Figure S1B. Decay constant distributions comparing proliferating (red) versus 7-day contact inhibited fibroblasts (green) separated by k-means clusters. Figure S2A. Gene set enrichment analysis of genes destabilized with quiescence. Figure S2B. Gene set enrichment analysis of genes stabilized with quiescence (2.7 mb). (PDF 2619 kb) [file 12864_2017_3521_MOESM3_ESM.pdf]
